# Grad-CAM Enabled Breast Cancer Classification with a 3D Inception-ResNet V2: Empowering Radiologists with Explainable Insights

**DOI:** 10.3390/cancers16213668

**Published:** 2024-10-30

**Authors:** Fatma M. Talaat, Samah A. Gamel, Rana Mohamed El-Balka, Mohamed Shehata, Hanaa ZainEldin

**Affiliations:** 1 Faculty of Artificial Intelligence, Kafrelsheikh University, Kafrelsheikh 33511, Egypt; fatma.nada@ai.kfs.edu.eg; 2 Faculty of Computer Science & Engineering, New Mansoura University, Gamasa 35712, Egypt; 3 Electronics and Communication Engineering Department, Faculty of Engineering, Horus University Egypt, Damietta 34518, Egypt; sgamel@horus.edu.eg; 4 Computers and Control Systems Engineering Department, Faculty of Engineering, Mansoura University, Mansoura 35516, Egypt; rannamohamedelbalka@std.mans.edu.eg (R.M.E.-B.); eng_hanaa@mans.edu.eg (H.Z.); 5 Electronic Engineering Department Higher, Institute of Engineering and Technology, Manzala 35642, Egypt; 6 Department of Bioengineering, Speed School of Engineering, University of Louisville, Louisville, KY 40292, USA

**Keywords:** breast cancer diagnosis, BCaXAI, deep learning, mammogram imaging, Grad-CAM, explainable AI

## Abstract

Breast cancer (BCa) poses a severe threat to women’s health worldwide as it is the most frequently diagnosed type of cancer and the primary cause of death for female patients. The biopsy procedure remains the gold standard for accurate and effective diagnosis of BCa. However, its adverse effects, such as invasiveness, bleeding, infection, and reporting time, keep this procedure as a last resort for diagnosis. A mammogram is considered the routine noninvasive imaging-based procedure for diagnosing BCa, mitigating the need for biopsies; however, it might be prone to subjectivity depending on the radiologist’s experience. Therefore, we propose a novel, mammogram image-based BCa explainable AI (BCaXAI) model with a deep learning-based framework for precise, noninvasive, objective, and timely manner diagnosis of BCa. The proposed BCaXAI leverages the Inception-ResNet V2 architecture, where the integration of explainable AI components, such as Grad-CAM, provides radiologists with valuable visual insights into the model’s decision-making process, fostering trust and confidence in the AI-based system. Based on using the DDSM and CBIS-DDSM mammogram datasets, BCaXAI achieved exceptional performance, surpassing traditional models such as ResNet50 and VGG16. The model demonstrated superior accuracy (98.53%), recall (98.53%), precision (98.40%), F1-score (98.43%), and AUROC (0.9933), highlighting its effectiveness in distinguishing between benign and malignant cases. These promising results could alleviate the diagnostic subjectivity that might arise as a result of the experience-variability between different radiologists, as well as minimize the need for repetitive biopsy procedures.

## Simple Summary

A novel mammogram image-based BCa explainable AI (BCaXAI) model is proposed, with a deep learning-based framework, for precise, noninvasive, objective, and timely manner diagnosis of BCa. The proposed BCaXAI leverages the Inception-ResNet v2 architecture, where the integration of explainable AI components, such as Grad-CAM, provides radiologists with valuable visual insights into the model’s decision-making process, fostering trust and confidence in the AI-based system.

## 1. Introduction

Breast cancer (BCa) is a significant global health issue, affecting millions of women worldwide. According to the World Health Organization (WHO), it is the second most common type of cancer after lung cancer. In 2022, approximately 2.3 million women were diagnosed with BCa, and 670,000 deaths were attributed to the disease [1]. While BCa can affect women at any age after adolescence, its prevalence increases with age [1]. In the United States, one in eight women will develop BCa. According to the American Cancer Society, in the United States, breast cancer remains a significant health concern, with approximately 310,720 new cases and 42,250 deaths expected in 2024 [2]. Additionally, in 2024, there were approximately 281,550 new cases and 43,600 deaths [3]. The WHO Global Breast Cancer Initiative (GBCI) aims to reduce the global BCa death rate by 2.5% annually between 2020 and 2040, preventing 2.5 million BCa deaths worldwide. Achieving this goal requires three key components: early detection through health prevention, rapid diagnosis, and comprehensive BCa.

BCa is characterized by excessive cell proliferation in the breast tissue, leading to the formation of masses or tumors. These tumors can be benign (non-cancerous) or malignant (cancerous). While benign tumors generally do not spread to other tissues and may not require treatment, malignant tumors can be invasive and metastasize to other organs, such as the liver, brain, bones, or lungs [4,5,6,7].

One or more approaches can be used to diagnose cancer, such as physical examination, laboratory tests, imaging tests, and biopsy. Choosing which biopsy procedure is right for you depends on the type of cancer and its location. In most situations, a biopsy is the only way to definitively diagnose cancer, but it takes a longer time to give results from a biopsy, in addition to some post-biopsy side effects such as infection, bleeding, lingering pain, redness, or swelling of the biopsy site [8,9]. Therefore, it is resorted to scans (such as a mammogram). Mammography is a widely used imaging technique for BCa diagnosis. There are two primary types of mammograms: digital mammography (DM) or 2D mammography and digital breast tomosynthesis (DBT) or 3D mammography [10]. DBT is becoming the preferred method due to its ability to provide more detailed images and reduce the need for additional views [11,12].

While mammograms are essential for BCa detection, their interpretation can be challenging and time-consuming, requiring specialized expertise. Computer-assisted diagnostic (CAD) systems offer a promising solution by aiding radiologists in identifying abnormalities and suspicious areas in mammograms. These systems utilize image processing techniques to analyze mammograms and provide support for diagnosis, tumor classification, and grading [13,14]. Convolutional neural networks (CNNs) have emerged as a powerful tool for BCa detection and classification in mammograms. CNNs are deep learning (DL) architectures that can automatically extract features from images, making them well-suited for medical image analysis. However, the black-box nature of DL models can raise concerns about interpretability and trustworthiness, particularly in critical applications like cancer detection [15,16,17,18].

While significant progress has been made in BCa detection using DL algorithms, research gaps still exist in previous BCa detection algorithms:

I. Limited interpretability: Many DL models used for BCa detection are considered black boxes, making it difficult to understand their decision-making process.
II. Data imbalance: with a higher prevalence of benign cases compared to malignant ones. This can bias models toward predicting benign cases, leading to missed detections.
III. Generalizability: DL models trained on specific datasets may not generalize well to unseen data.
IV. Robustness to noise: Mammograms can be affected by noise due to factors like image artifacts, patient positioning, and equipment variations.
V. Integration with clinical workflows: Integrating DL models into existing clinical workflows can be challenging due to factors such as computational requirements, user interface design, and regulatory considerations.
VI. Explainable AI (XAI): Needed to develop methods that can provide clear and meaningful explanations for DL models in the context of BCa detection.

Addressing these research gaps is crucial for developing more reliable, interpretable, and clinically applicable DL models for BCa detection. Therefore, there is a pressing need for a BCa classification model that:

- Achieves high accuracy and sensitivity to ensure early detection of BCa.
- Provides explainable insights to enhance trust and transparency in the diagnostic process.
- It is robust and generalizable to various mammogram datasets and imaging modalities.
- It can be integrated into clinical workflows for practical application.

In this paper, we propose a novel approach combining the 3D Inception-ResNet V2 architecture with gradient-weighted class activation mapping (Grad-CAM) for BCa classification. This approach provides explainable insights by highlighting the regions of the mammogram that contribute most to the model’s predictions, enhancing the trust and transparency of the diagnostic process.

The main contributions of this paper can be summarized as follows:

- Developed BCaXAI, a novel explainable AI model for BCa classification.
- Utilized the Inception-ResNet V2 architecture for superior performance.
- Achieved high accuracy, recall, precision, F1-score, and AUROC.
- Incorporated XAI components for transparency and interpretability.
- Conducted extensive evaluation of DDSM and CBIS-DDSM datasets.
- Outperformed traditional models (ResNet50, VGG16).
- Demonstrated potential as a valuable tool for clinical practice.
- Proposed future research directions for model validation and expansion.

BCaXAI distinguishes itself from existing AI models for breast cancer diagnosis in the following ways:

i. Explainability through XAI integration:

While many AI models function as “black boxes,” BCaXAI integrates Grad-CAM visual explanations to provide heatmaps that highlight key image regions influencing predictions. This feature enhances model transparency, fostering trust among clinicians and patients.

ii. Patient-centered interpretability:

Unlike traditional models that prioritize predictive accuracy alone, BCaXAI emphasizes interpretability, allowing healthcare professionals to understand why a particular diagnosis is made and facilitating better decision-making and discussions with patients.

iii. Robustness and reliability:

BCaXAI incorporates advanced mechanisms, such as attention modules, to focus on subtle patterns within images, improving its performance in challenging cases like dense breast tissue or ambiguous lesions. This targeted feature ensures fewer false positives or negatives compared to conventional models.

iv. Comprehensive dataset utilization:

Our model was trained and validated on a well-curated, diverse dataset to avoid bias and generalization issues. It ensures adaptability across varying patient demographics, which is not always the case with existing AI systems.

v. End-to-End workflow suitability:

BCaXAI not only assists with diagnosis but also integrates smoothly into clinical workflows by providing visual justifications. This reduces reliance solely on radiologist intuition, bridging the gap between AI predictions and human expertise.

These features position BCaXAI as a more transparent, reliable, and patient-centric tool compared to many existing black-box AI models in breast cancer diagnosis.

The rest of the paper is organized as follows: A brief review of state-of-the-art techniques introduced for BCa detection is discussed in Section 2. The data used and the proposed technique are described in detail in Section 3. Section 4 depicts the evaluation of the proposed model and experimental results. Discussion and conclusions are presented in Section 5.

## 2. Literature Review

### 2.1. DL Architectures for BCa Classification

ResNet, VGG, and AlexNet Performance: The study by [19] explored ResNet50, VGG16, and AlexNet for classifying mammographic images into normal, benign, and malignant categories, with ResNet50 achieving the highest accuracy (96.23%). Similarly, [20] this study evaluates pre-trained deep transfer learning models, including ResNet50 and VGG variants, for BCa detection using a dataset of 2,453 histopathological images. Results indicate that ResNet50 outperforms other models, achieving an accuracy of 92.2%. Fine-tuning and transfer learning: Research in [21] focused on fine-tuning ResNet50V2 using ultrasound images, achieving 99.11% accuracy. This highlights the effectiveness of transfer learning when tailored to the task-specific dataset. In [22], the authors utilized pre-processed mammogram images to detect BCa. The generated data are then input into three CNNs with perceptual variations. In the following approach, the identical set of photos is fed into pre-trained VGG19, ResNet50, MobileNet-V2, Inception-V3, Xception, and Inception-ResNet-V2 via transfer learning via fine-tuning.

### 2.2. Attention Mechanisms and Lightweight Models

Attention-based models: In [23], a lightweight attention-based ResNet50 model addressed data imbalance, leading to impressive performance (98.5%-98.7% accuracy) on the BreakHis dataset by focusing on informative regions in histopathological images. Ensemble approaches: The CAD system proposed in [24], using a stacked ensemble of ResNet models, outperformed individual models, particularly in pathology classification and BI-RADS categorization.

### 2.3. Federated Learning and Privacy Concerns

Privacy-preserving techniques: The work in [25] introduced federated learning for BCa diagnosis using the Wisconsin Diagnostic Breast Cancer Dataset. This method maintained high accuracy (97.59%) while addressing privacy concerns, crucial for handling sensitive medical data.

### 2.4. Weakly Supervised and 3D DL Techniques

Lesion localization and 3D approaches: In [26], weakly supervised 3D DL techniques were employed for lesion localization and classification using MRI data. The researchers fixed an erratum that provided more accurate performance baselines for radiologists.

### 2.5. Segmentation and Visualization

U-Net for segmentation: The study [27] employed U-Net for BCa segmentation, achieving superior performance with an IoU score of 84.60% and an F1-score of 83.70%. Grad-CAM visualization techniques were used to enhance interpretability. Image Augmentation and enhancement: In [28], the authors combined U-Net-based segmentation with image enhancement and data augmentation to improve classification accuracy, achieving 86.71% accuracy and 91.34% recall.

### 2.6. Hybrid and Feature Engineering Approaches

Hybrid CNN approaches: Paper [29] proposed combining hand-crafted features like local binary pattern (LBP) and histogram of oriented gradients (HOG) with CNN-extracted features, integrating domain expertise for better performance. EACO-ResNet hybrid models: In [30], the EACO-ResNet101 model showed strong performance on the CBIS-DDSM dataset with 98.63% accuracy, emphasizing the value of hybrid techniques.

While significant progress has been made in DL for BCa detection, further research is needed to address challenges such as interpretability, data imbalance, and generalizability. By combining advanced DL techniques with explainable AI and addressing these research gaps, we can develop more reliable and clinically applicable tools for early detection and accurate diagnosis of BCa.

## 3. Materials and Methods

This section describes detailed information about the data used and the proposed model with detailed step descriptions, including data preprocessing, model architecture, and the experimental setup, including data splitting, training parameters, and validation procedures.

### 3.1. Dataset Description

In this study, we utilized two widely recognized mammography datasets: The Curated Breast Imaging Subset of the Digital Database for Screening Mammography (CBIS-DDSM) [31] and the broader Digital Database for Screening Mammography (DDSM). These datasets contain high-quality, annotated mammographic images that have been instrumental in developing and testing machine-learning models for breast cancer screening and diagnosis.

CBIS-DDSM, curated from the larger DDSM database, offers enhanced image quality and standardization, making it particularly valuable for research on early detection of breast cancer. The images, provided in DICOM format, include comprehensive annotations by radiologists, covering breast density, lesion types (e.g., masses and calcifications), and pathology data (benign vs. malignant). These annotations are crucial for tasks such as breast density calculation, lesion segmentation, and classification.

We ensured a balanced representation of benign and malignant cases for training and validation to address any potential dataset generalizability issues. Thanks to the varied annotations and the structured nature of CBIS-DDSM, we established a strong validation procedure by testing the model on a different, independent set to precisely evaluate performance. This well-balanced dataset, which includes comprehensive region-of-interest (ROI) marks, aids in creating models meant to increase the precision of breast cancer diagnosis in clinical settings.

It is worth noting that pathology reports are available for the majority of cases in the dataset, ensuring the reliability and accuracy of the ground truth labels. However, there are a few cases where pathology reports are either missing or incomplete. In such instances, additional metadata—such as radiology reports and clinical notes—were utilized to complement the diagnosis and mitigate the impact of these gaps.

To maintain data integrity, the following strategies were implemented:

1. Case exclusion criteria: Cases with significant missing pathology information were excluded from training to prevent introducing noise or ambiguity in the model.

2. Validation with clinical metadata: Where minor gaps existed (e.g., incomplete pathology descriptions), cross-validation with clinical metadata helped ensure the consistency and reliability of labels.

3. Dataset partitioning: Cases with complete pathology reports were prioritized for the test and validation sets to guarantee robust model evaluation.

By taking these steps, we ensured that the dataset remains comprehensive, with minimal gaps that would affect the model’s performance or the validity of our results.

**Figure 1:**
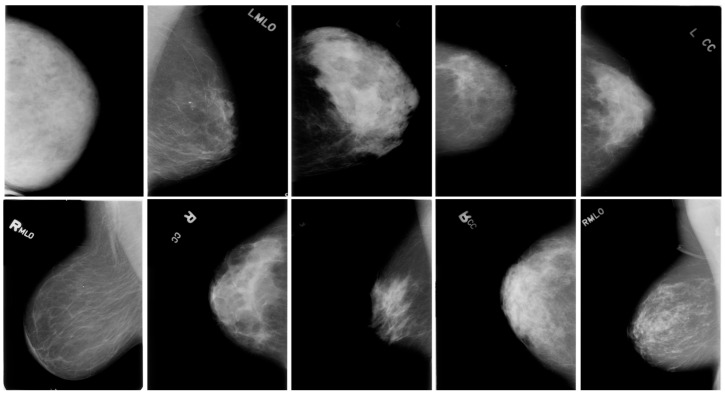
Example of dataset images.

A dataset example is shown in Figure 1.

The DDSM-CBIS dataset states that the data were separated into test and training sets. The dataset contains both positive and negative state images from the CBIS-DDSM dataset and the DDSM dataset. The data were pre-processed to create 299 × 299 images. Before being downsized to 299 × 299 pixels, the negative images from the DDSM dataset were tiled into the size 598 × 598 pixels before being resized to the size 299 × 299.

The ROIs for the positive images of the CBIS-DDSM dataset were extracted depending on using masks with padding of small values to produce context. Each ROI was then cropped randomly three times into an image size of 598 × 598 pixels, after that, random rotations and flips, before being resized to images with size 299 × 299 pixels.

### 3.2. Breast Cancer Explainable Artificial Intelligence (BCaXAI)

**Figure 2:**
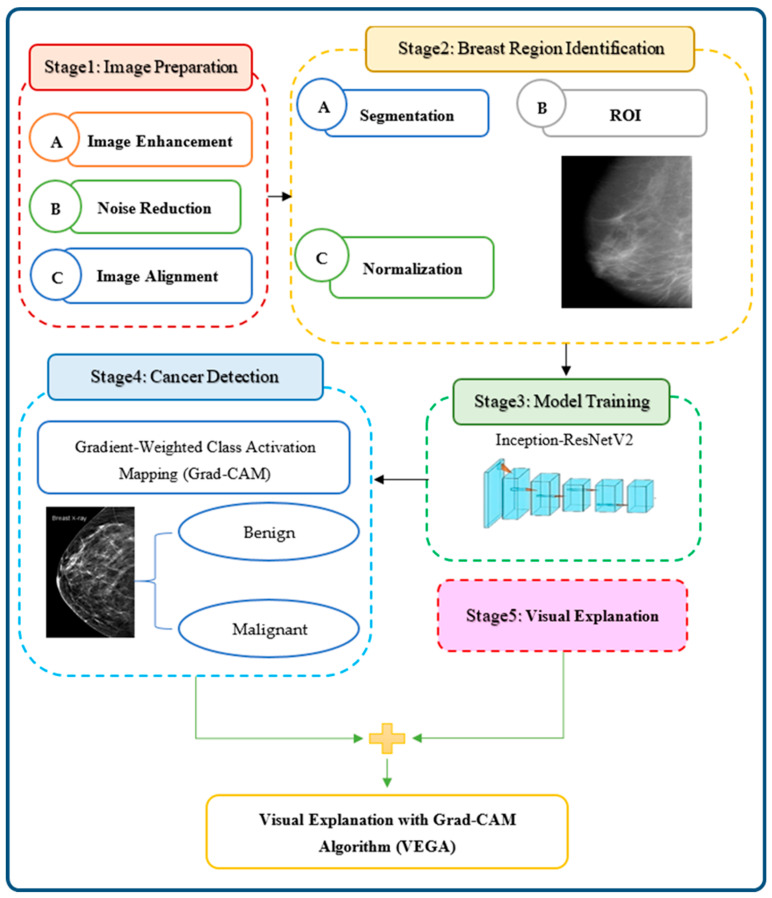
The proposed BCaXAI stages will have detailed descriptions, including image preparation, breast region identification, classification model training, cancer detection, and visual explanation.

BCaXAI is an advanced algorithm designed to improve BCa diagnosis through the analysis of 3D mammograms. By enhancing sophisticated image processing techniques, cutting-edge DL models, and interpretable AI, BCaXAI offers a comprehensive approach to enhancing diagnostic accuracy and efficiency. The process involves several stages, as illustrated in Figure 2. By combining advanced image processing, DL, and interpretable AI, BCaXAI aims to improve the accuracy and efficiency of BCa diagnosis.

#### 3.2.1. Image Preparation

The initial stage of BCaXAI involves preparing mammogram images for analysis. This crucial step aims to enhance image quality and consistency. Several techniques are employed (image enhancement-noise reduction-image alignment) to improve image clarity by optimizing contrast and brightness levels, eliminating unwanted image disturbances, such as graininess, and standardizing image orientation and size.

Steps for image preparation/preprocessing of input images are detailed in Algorithm 1.
**Algorithm 1: Image Preparation Algorithm (IPA)**Input: Raw mammogram image dataset (Ds)Output: Preprocessed mammogram image dataset (Dsp)Steps:Initialization: Create an empty list (Dsp) to store processed images.Image Loop: Process each image (Img) in the dataset (Ds):Enhancement: Increase image contrast and apply histogram equalization to improve visibility of details.Noise Reduction: Reduce image noise using Gaussian blur and median filtering to enhance clarity.Alignment: Detect key points in the image and apply geometric transformations to ensure consistent image orientation.○Histogram Equalization:✓Calculate the cumulative distribution function (CDF) of the image histogram.✓Map pixel intensities to new values based on the CDF.○Gaussian Blur:✓Apply a Gaussian kernel to the image, defined by: $G\left(x,\;y\right)=\left(\frac{1}{2*pi*sigma^{2}}\right)*\;exp\left(-\frac{x^{2}+y^{2}}{2*sigma^{2}}\right)$✓Convolve the image with the Gaussian kernel.✓Median Filter:✓Replace each pixel with the median value of its neighboring pixels.6.Storage: Add the processed image to the output list (Dsp).7.Output: Return the preprocessed image dataset (Dsp).

#### 3.2.2. Breast Region Identification

The second phase of the BCaXAI system involves precisely locating the breast region within the preprocessed mammogram images.

Segmentation is a critical step in our BCaXAI framework, as it involves isolating the breast region from the surrounding background in mammogram images. This process enhances the model’s ability to focus on relevant features, such as lesions or masses, which are vital for accurate diagnosis. In our approach, we utilized advanced segmentation techniques, including thresholding and morphological operations, to delineate the breast area precisely. This segmentation output was then incorporated into the classification model by serving as the input images for the Inception-ResNet V2 architecture, ensuring that only the most relevant regions of interest (ROIs) were analyzed. Rather than employing a voting method for features, we utilized a direct integration of the segmented images into the classifier, which allowed the model to learn from the essential features of the breast tissue more effectively. This method not only improved classification performance but also reduced false positives by minimizing the influence of irrelevant background noise, thereby enhancing the overall diagnostic accuracy of the system. This step is crucial for optimizing computational efficiency and enhancing the accuracy of subsequent analysis. Algorithm 2 outlines the breast region identification algorithm (BRIA). Several image processing techniques are employed to achieve (segmentation-region of interest-normalization), which isolates the breast area from the surrounding background and irrelevant elements and identifies specific areas within the breast region that are most likely to contain diagnostic information. In addition, the normalization standardizes the size and orientation of the identified breast region for consistent analysis.
**Algorithm 2: Breast Region Identification Algorithm (BRIA)**Input: Preprocessed mammogram image dataset (Dsp)Output: Dataset of images with identified breast regions (Dsr)Steps:1.Initialization:-Create an empty list (Dsr) to store images with identified breast regions.2.Image Loop:-For each image (Img) in the dataset (Dsp):a.Segmentation:-Apply thresholding to convert the image to a binary format, isolating the breast region.-Use edge detection methods to refine the boundary of the breast region.b.ROI Detection:-Apply morphological operations to enhance the identified regions.-Use contour detection to locate the regions of interest (ROIs) within the breast region.c.Normalization:-Align the breast region using key anatomical landmarks.-Scale the breast region to a standard size for uniform analysis.-Crop the image to focus on the breast region.d.Storage:-Add the image with the identified breast region to the output list (Dsr).3.Output:-Return the dataset of images with identified breast regions (Dsr).

**Figure 3:**
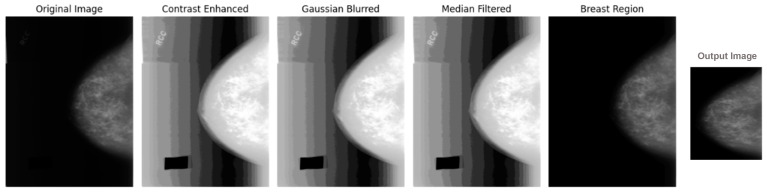
An illustrative example showing the steps applied, starting with an original image going through the preprocessing algorithm with its different steps, including contrast enhancing through histogram equalization and bias correction, noise reduction, and smoothing through Gaussian blurring and median filtering. Then, the final breast region of interest (normalized image) will be identified and fed to the DL model.

Through these straightforward procedures, the breast region identification phase ensures image readiness for the DL model, ultimately improving the diagnostic accuracy of BCaXAI. Figure 3 represents an illustrative example of an image being preprocessed till obtaining the identified breast region of interest as an output from BRIA.

#### 3.2.3. Model Training

**Figure 4:**
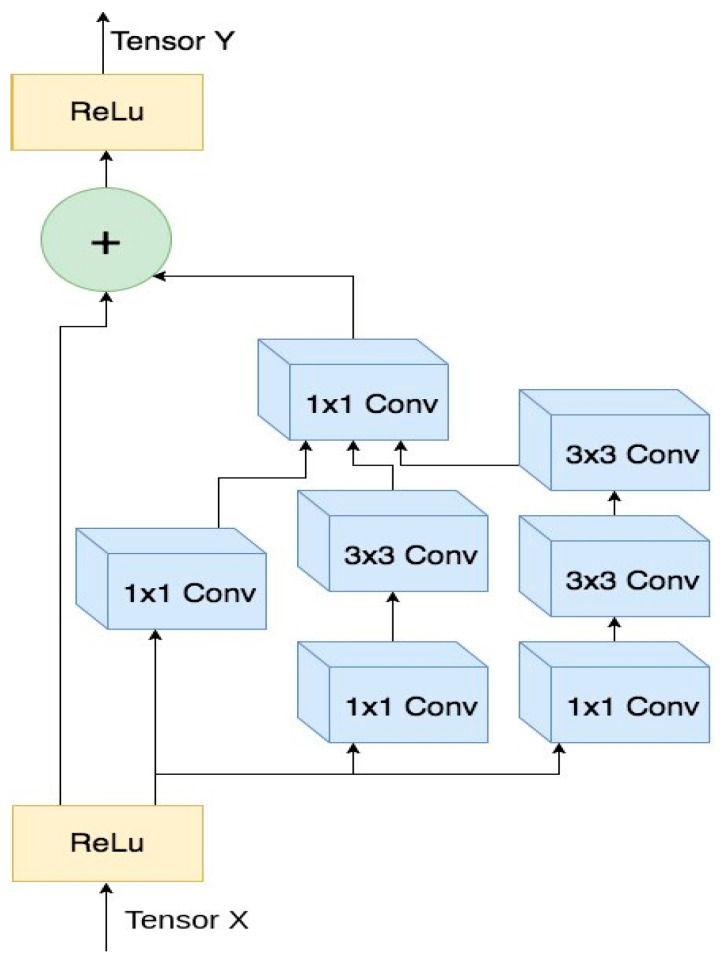
Modified Inception-ResNetV2 architecture.

This phase incorporates a modified Inception-ResNetV2 architecture. The original Inception-ResNetV2 model employs a multi-layered structure where subsequent layers are interconnected via multiple nodes undergoing nonlinear transformations. Optimal performance hinges on the meticulous allocation of weights, biases, activation functions, loss models, and optimization parameters. While bidirectional neural networks predominate in many DL models, the current approach utilizes a feedforward architecture. Figure 4 illustrates the modified Inception-ResNetV2’s core structure.

To enhance model performance, the ReLU activation function has been replaced with the Hard Swish function. Inspired by the Swish function, Hard Swish introduces a computationally efficient linear approximation in place of the computationally demanding sigmoid function, as detailed in Equation (1) [24].

(1) $$h-swich\left(x\right)=x\frac{RelU6\left(x+3\right)}{6}$$

#### 3.2.4. Cancer Detection

The final stage of the BCaXAI system involves classifying BCa images into benign or malignant categories. Algorithm 3 outlines the Grad-CAM-based BCaXAI model for this classification task.
**Algorithm 3: Grad-CAM Based Breast Cancer Classification Algorithm**Input: Dataset images (Ds)Output: Image target for target labels (j = 0, 1, 3, …)Steps:Data Augmentation: Enhance data diversity through augmentation techniques.Gradient Calculation: Compute the gradient of the target class concerning feature map activations of a convolutional layer.Weight Calculation: Calculate weights (alphas) by averaging gradients.Grad-CAM Heatmap Generation: Create a heatmap using calculated weights and feature maps.Data Splitting: Divide the dataset into training (80%), validation (10%), and testing (10%) sets.Model Training: Train the Inception-ResNet(50) pre-trained model on the training data and save the resulting model (Nk).○Augmentation process○Computing the gradient of $y_{c}$ with respect to the feature map activations $A^{C}$ of a convolutional layer, i.e., $\frac{\partial y_{c}}{\partial A^{c}}$○Calculating Alphas by Averaging Gradients: $\propto_{k}^{c}=\frac{1}{z}\underset{i}{\sum }\underset{j}{\sum }\frac{\partial y^{c}}{\partial A_{ij}^{k}}$○Calculate Final Grad-CAM Heat map: $L_{Grad-CAM}^{c}=ReLU\left(\underset{k}{\sum }\propto_{k}^{c}A^{k}\right)$○Inception-Resnet (50) pre-trained model on training data○Save the BDM model Nk7.Model Evaluation: Evaluate the saved model (Nk) on the test data.8.Prediction: Generate the final prediction based on the model’s output.

This algorithm leverages the power of DL, specifically the Inception-ResNetV2 architecture, combined with the Grad-CAM technique to accurately classify BCa images.

#### 3.2.5. Visual Explanation

Grad-CAM, Grad-CAM++, and Score-CAM are all visualization techniques used to interpret the decisions of CNNs, particularly in tasks like image classification.

Grad-CAM employs gradients of the target class flowing into the last convolutional layer of a CNN (i.e., the class of interest, such as a particular form of cancer in breast cancer diagnosis). A coarse localization map that highlights significant areas of the image is computed using these gradients. It can easily be implemented and works well across different models. It is class-specific, meaning it can visualize the contribution of a particular class to the decision-making. However, sometimes, it may produce coarser, less accurate heatmaps that miss finer elements in the image.

An enhanced version of Grad-CAM is called Grad-CAM++. With a more precise determination of the significance of every pixel, it computes weighted averages of the gradients. Additionally, it considers higher-order terms in the gradient, which increases its sensitivity to the pixel positions in space. It generates heatmaps with greater detail and sharpness than Grad-CAM, particularly in situations where the categorization may be influenced by several objects or areas. It works especially well with photos that include overlapping items. However, it may take longer to compute and requires more computing power than Grad-CAM.

Score-CAM does not use gradients at all. Rather, it creates a heatmap by applying a mask to certain areas of the image and then running the masked images through the model to determine the extent to which the mask affects the final score. These regions are combined in a weighted manner according to their influence to create the final heatmap. The main advantage of Score-CAM is that it is less dependent on gradients, which can occasionally be loud or erratic, making it more resilient. Makes heat maps that are more precise and readable. However, each masked region requires numerous forward runs through the model; it is significantly more computationally costly.

We have considered Grad-CAM with the proposed BCaXAI system, as it is good for initial insights, offering a balance between interpretability and speed. It can give a general idea of which regions are important (tumor regions).

To enhance transparency and trust in the BCaXAI system, a visual explanation phase is incorporated. This aims to elucidate the model’s decision-making process by highlighting the key image regions influencing its predictions. Grad-CAM, a gradient-based visualization technique, is employed to generate heatmaps superimposed on the original mammograms. These heatmaps accentuate areas critical to the model’s classification, enabling radiologists to correlate the AI’s findings with their own expert knowledge, thereby fostering trust and confidence in the system’s diagnostic capabilities. The overall steps of the visual explanation with Grad-CAM algorithm (VEGA) are illustrated in Algorithm 4, where pre-processed mammogram images (Dsp), trained model (Nk), and class activation maps (CAM) are input to arrive at visual interpretations with heatmaps overlaid on the mammogram images.
**Algorithm 4: Visual Explanation with Grad-CAM Algorithm (VEGA)**Input: Preprocessed mammogram images, Trained model, Class activation mapsOutput: Visual explanations with heatmaps overlaid on mammogramsSteps: 1.Initialize: -Load the preprocessed mammogram images. -Load the trained model. 2.Loop through images dataset: For each image in preprocessed mammogram images:
 a.Model Prediction: -Use the trained model to predict the image class. b.Gradient Computation: -Compute gradients of the predicted class concerning the feature maps of the last convolutional layer. -Gradients = ∂ (Predicted Class)/∂ (Feature Maps)  c.Weight Calculation: -Average the gradients over all spatial locations to obtain weights (α). -α_k_ = 1/Z * Σ_i_ Σ_j_ (∂(Predicted Class)/∂(Feature Maps_ij_))  d.Grad-CAM Heatmap Generation: -Compute the weighted sum of the feature maps using the calculated weights (α). -Heatmap = ReLU(Σ_k_ (α_k_ * Feature Maps_k_)) -Normalize the heatmap to the range (0, 1) for visualization.  e.Overlay Heatmap: -Resize the heatmap to match the original image’s dimensions. -Superimpose the heatmap on the original mammogram image to highlight areas of importance. f.Display and Save: -Display the overlaid heatmap along with the original image. -Save the visual explanation for further review.

Grad-CAM visualizations play a critical role in enhancing radiologists’ trust and understanding of AI-assisted diagnostics by providing a transparent, interpretable layer of insight into the decision-making process of deep learning models. Specifically:

i. Highlighting clinically relevant features: Grad-CAM generates heat maps that indicate the areas of the medical images that contribute most to the model’s predictions. This alignment between the model’s focus and clinically relevant regions, such as suspicious lesions or masses, gives radiologists confidence that the AI is not making predictions based on irrelevant information.
ii. Promoting collaborative decision-making: By offering a visual explanation, Grad-CAM helps radiologists better understand the rationale behind AI recommendations. When AI outputs align with their clinical reasoning, it reinforces trust. In cases of disagreement, it prompts further investigation and discussion, fostering collaborative decision-making.
iii. Reducing the black-box nature of AI models: One of the biggest challenges in AI adoption is the lack of transparency in deep learning models. Grad-CAM transforms abstract neural network outputs into visual explanations, bridging the gap between AI and human understanding.
iv. Facilitating model validation and error analysis: When radiologists can see how the model arrives at a particular conclusion, they are better equipped to identify potential errors or biases, improving both the model’s reliability and their own confidence in using it.

By following these steps, VEGA provides valuable insights into the model’s decision-making process, aiding radiologists in interpreting the AI’s predictions.

BCaXAI offers a comprehensive approach to enhancing BCa diagnosis by combining advanced image processing techniques, DL models, and interpretable AI. The key contributions of BCaXAI include:

- Improved Diagnostic Accuracy: BCaXAI leverages a sophisticated DL model, Inception-ResNetV2, to accurately classify BCa images.
- Enhanced explainability: The integration of Grad-CAM, Grad-CAM++, and Score-CAM heatmaps provide visual explanations of the model’s decision-making process, fostering trust and transparency.
- Robustness and efficiency: The algorithm incorporates efficient image processing techniques and optimization strategies to ensure robustness and computational efficiency.
- Clinical applicability: BCaXAI has the potential to be integrated into clinical workflows, aiding radiologists in making more accurate and informed diagnoses.

## 4. Experimental Results

Using Inception-ResNet v2, the suggested framework is trained on the CBIS-DDSM [31] dataset. Every architecture’s prediction performance is assessed using several evaluation metrics.

### 4.1. BCaXAI Evaluation

The performance metrics of the BCaXAI model, as presented in Table 1, demonstrate exceptional capabilities in BCa classification. The model achieved an outstanding accuracy of 98.53%, indicating a high degree of correct predictions. This high accuracy rate is crucial for clinical applications as it minimizes the risk of misdiagnosis.

Furthermore, the model exhibited a recall of 98.53%, suggesting its ability to effectively identify a substantial proportion of positive cases. This is particularly important in BCa diagnosis as early detection is critical for successful treatment. The F1-score of 0.9843 and precision of 0.984 demonstrate a robust balance between precision and recall, indicating that the model not only identifies a high proportion of positive cases but also minimizes false positives.

The area under the receiver operating characteristic curve (AUROC) score of 0.9933 is exceptionally high, signifying the model’s excellent discriminative power. This metric highlights the model’s ability to differentiate between benign and malignant cases with high confidence.

Overall, the BCaXAI model’s performance metrics are highly promising and suggest its potential as a valuable tool in assisting radiologists in BCa diagnosis. These results underscore the efficacy of the proposed approach and warrant further investigation for clinical implementation.

### 4.2. Comparison against Benchmark DL Models

As illustrated in Table 2, the proposed BCaXAI model significantly outperforms both ResNet50 and VGG16 across all evaluated metrics. This superiority is particularly evident in accuracy, recall, precision, F1-score, and AUROC, demonstrating the model’s exceptional ability to accurately classify BCa cases. The substantial performance gains achieved by BCaXAI underscore the effectiveness of its architecture and the incorporation of XAI components in addressing the challenges posed by BCa diagnosis.

These results highlight the potential of BCaXAI as a valuable tool for radiologists to improve diagnostic accuracy and reduce the risk of misdiagnosis. By providing superior performance compared to established models, BCaXAI demonstrates its potential to enhance patient care and outcomes. Also, achieving higher accuracy and potentially improving the diagnostic capabilities of the system.

However, further research is warranted to validate these findings on larger and more diverse datasets, as well as to explore the clinical impact of the model in real-world settings. Additionally, investigating the generalizability of BCaXAI to other medical imaging tasks could broaden its applicability beyond BCa diagnosis.

### 4.3. Summary Analysis of the State-of-the-Art Approaches from Literature

Table 3 illustrates a comparative analysis of recent studies on DL techniques for BCa classification and diagnosis. This work highlights the potential of ensemble learning and the strategic combination of DL techniques to enhance the performance and reliability of computer-aided BCa diagnosis systems.

### 4.4. Comparing BCaXAI with the State-of-the-Art Approaches

Several cutting-edge BCa detection techniques are considered to evaluate how well the proposed BCaXAI model performs in contrast to other methods. The results of the comparison and the level of accuracy attained by each approach are shown in Table 4. A variety of evaluation criteria, including accuracy, recall, precision, and F1-score, are used to study and evaluate the performance of the suggested model. The evaluation results showed that the suggested model, based on the proposed BCaXAI, outperformed the other techniques. It was more accurate in identifying BCa. Regarding its real-time capabilities, the proposed BCaXAI model can effectively detect BCa from medical images.

The BCaXAI model demonstrated exceptional performance across all evaluation metrics, as highlighted in Table 4. The model achieved an accuracy of 98.53%, indicating a high degree of correct classifications. Additionally, the recall of 98.53% suggests that the model successfully identified a substantial portion of positive cases. The F1 score of 0.9843 and precision of 0.984 demonstrate a strong balance between precision and recall. Furthermore, an AUROC score of 0.9933 indicates excellent discriminative power, suggesting the model’s ability to effectively differentiate between positive and negative cases.

### 4.5. Explainability Using GRAD-CAM, Grad-CAM++, and Score-CAM Heatmaps Techniques

To understand the model’s decision-making process and build trust in its predictions, Grad-CAM, Grad-CAM++, and Score-CAM heatmaps were employed to generate visual explanations. The heatmaps generated by Grad-CAM, Grad-CAM++, and Score-CAM highlighted the regions of the mammogram images that contributed the most to the model’s classification decisions. By examining these heatmaps, radiologists could correlate the model’s predictions with their own expert knowledge, enhancing their understanding and confidence in the AI system’s findings.

Through visual inspection of the Grad-CAM, Grad-CAM++, and Score-CAM heatmaps, the model correctly identifies clinically significant areas, such as regions containing masses and calcifications. This alignment with radiologists’ annotations ensures that the model is focusing on medically relevant features rather than spurious correlations, thus increasing the confidence in its predictions.

For example, in cases of benign lesions, the Grad-CAM, Grad-CAM++, and Score-CAM heatmaps often highlighted dense areas of breast tissue without focusing on specific localized masses, which aligns with radiologists’ observations that dense tissue can sometimes mimic benign findings. In contrast, for malignant cases, the heatmaps consistently covered the areas corresponding to calcifications or masses, both of which are essential diagnostic features in breast cancer detection.

The model’s interpretability improves Grad-CAM, Grad-CAM++, and Score-CAM explainability, which allows radiologists to not only believe the model’s predictions but also comprehend the reasoning underlying them. There is potential for increasing diagnostic precision and, eventually, patient outcomes through the integration of clinical knowledge with AI-generated insights.

**Figure 5:**
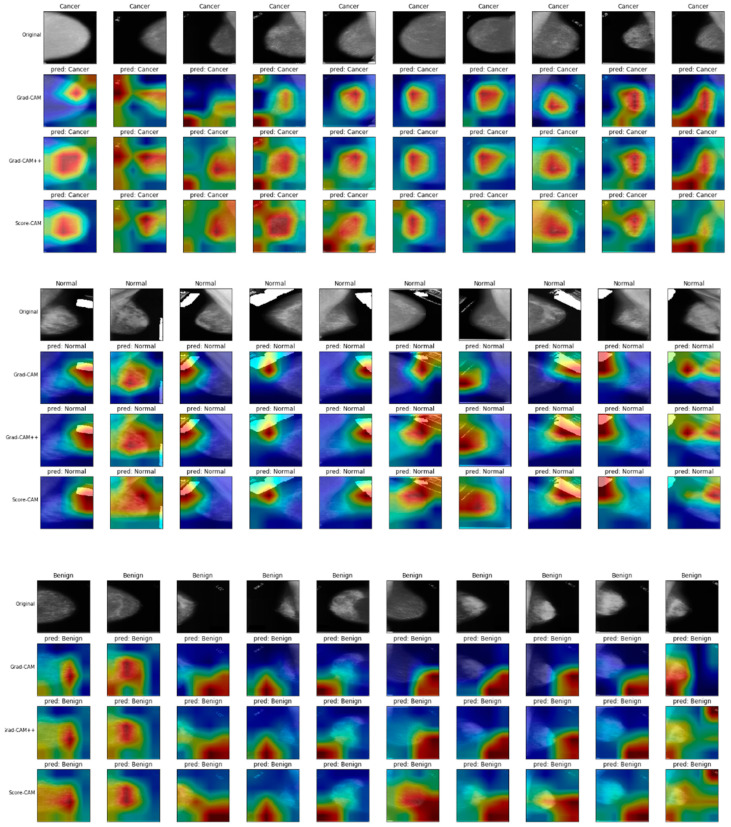
Comparison between Grad-CAM, Grad-CAM++, and Score-Cam for a subset of the testing dataset, including Malignant/cancerous cases, Normal, and Benign Cases. This color spectrum—from cool (blue) to hot (red)—visually highlights the regions of an image most responsible for the model’s prediction output, where the red and the blue represents the highest and the lowest activation areas, respectively.

Figure 5 displays examples of different malignant lesions and benign lesions with reference to normal lesions, along with their corresponding Grad-CAM, Grad-CAM++, and Score-CAM heatmaps. The heatmaps, generated using the BCaXAI model, highlight the regions of the mammogram images that contributed most to the model’s classification decisions. Warmer colors in the heatmaps indicate areas with a stronger impact on the model’s output, suggesting that the model focused on these regions when making its predictions. From this comparison between Grad-CAM, Grad-CAM++, and Score-Cam for a subset of the testing dataset, it is observed that Grad-CAM is often a good compromise between precision and computational cost for breast cancer detection, as it tends to produce a general idea of tumor regions and generate accurate heatmaps.

In a Grad-CAM heatmap, the colors typically represent the intensity of activation in different regions of an image. Here’s a standard color interpretation for such heatmaps:

- Red/Orange/Yellow: These colors usually represent high activation areas, indicating that the model focuses on these regions when making predictions.
- Red: Highest activation (critical areas for the prediction).
- Orange/Yellow: Moderate to high activation.
- Green: Intermediate activation level, suggesting some relevance but not as crucial as red/yellow regions.
- Blue: Low or no activation, meaning these regions have little influence on the model’s decision.
- Black/Dark blue: Areas of no activation or minimal importance for the prediction.

This color spectrum—from cool (blue) to hot (red)—visually highlights the regions of an image most responsible for the model’s output.

## 5. Discussion and Conclusions

This research introduced BCaXAI, a groundbreaking AI model that significantly advances BCa diagnosis by harnessing the power of DL and XAI. By seamlessly integrating the advanced 3D Inception-ResNet v2 architecture with Grad-CAM visualization techniques, BCaXAI offers a robust solution for accurately distinguishing between benign and malignant breast tumors. This integration not only enhances diagnostic accuracy but also provides critical insights into the model’s decision-making process, fostering greater trust and understanding among radiologists and clinicians.

Trained and validated on the extensive DDSM and CBIS-DDSM datasets, BCaXAI demonstrated exceptional performance, surpassing traditional models such as ResNet50 and VGG16 in multiple evaluation metrics. The model achieved a superior accuracy of 98.53%, alongside high recall (98.53%), precision (98.40%), F1-score (98.43%), and an impressive AUROC of 0.9933. These results underscore BCaXAI’s capability to significantly reduce false positives and false negatives, thus minimizing unnecessary biopsies and ensuring timely intervention for patients with malignant conditions.

Moreover, the use of Grad-CAM provides visual explanations of the model’s predictions, highlighting the specific regions of mammograms that influenced its decisions. This transparency is crucial for bridging the gap between automated AI diagnostics and clinical practice, empowering radiologists with actionable insights and reinforcing their confidence in AI-assisted decision-making. By mitigating the inherent subjectivity and variability in manual mammogram interpretation, BCaXAI not only streamlines the diagnostic workflow but also contributes to more consistent and reliable outcomes.

BCaXAI has the potential to be highly useful for other medical imaging tasks and diseases beyond breast cancer diagnosis for several reasons:

- Generalizability of Grad-CAM for interpretability:

The Grad-CAM visual explanation technique used in BCaXAI is not limited to breast cancer diagnosis. It can be applied across various medical imaging modalities—such as CT scans, MRIs, X-rays, and ultrasounds—to provide heatmaps highlighting critical regions influencing model predictions. This interpretability can enhance trust in AI models for tasks like detecting lung diseases (e.g., pneumonia or COVID-19), brain tumors, or cardiovascular abnormalities.

- Adaptability to multi-class classification tasks:

Just as BCaXAI handles different breast cancer subtypes, the underlying architecture can be adapted to multi-class classification problems in other areas of medical imaging, such as skin lesion classification (e.g., melanoma vs. benign lesions) or identifying retinal diseases.

- Improving diagnostics for complex cases:

Similar to breast cancer diagnosis, many diseases (e.g., Alzheimer’s, liver cirrhosis, or chronic lung diseases) require precise localization of abnormalities within imaging data. The transparent nature of BCaXAI’s visual outputs can assist healthcare professionals by ensuring the AI’s focus aligns with known pathology, improving diagnostic accuracy in complex cases.

- Integration with multi-modal data:

BCaXAI’s explainable approach can also be extended to medical models that rely on multi-modal data—combining images with clinical, genomic, or pathological reports. This could prove beneficial for diseases requiring comprehensive assessments, such as cancer recurrence prediction or patient prognosis estimation.

- Enhanced training and acceptance across medical specialties:

As BCaXAI emphasizes both accuracy and interpretability, it can help other specialties train practitioners to better understand AI outputs, encouraging broader acceptance of AI tools across different medical fields. For instance, radiologists working in neurology, cardiology, and pulmonology could benefit from similar visualization techniques to support clinical decision-making.

## 6. Limitations of the Study

While our study demonstrates the potential of the BCaXAI model for breast cancer diagnosis, it is essential to acknowledge certain limitations that may affect the generalizability of our findings.

One significant limitation is the reliance on the CBIS-DDSM dataset, which was created from film-based mammography images and subsequently digitized using a specific digitizer with approximately 50 µm pixel pitch. This method of image acquisition, which reflects technology that was prevalent several decades ago, contrasts with modern advancements in mammography imaging, such as full-field digital mammography (FFDM). The evolution of imaging technology has led to notable improvements in image quality, resolution, and diagnostic capabilities. As a result, the characteristics of the images in the CBIS-DDSM dataset may not accurately represent those obtained from contemporary FFDM systems.

The implications of this limitation are twofold. Firstly, while our model performs well on the CBIS-DDSM dataset, its performance on more recent, higher-resolution images remains to be validated. There may be differences in image artifacts, contrast, and overall quality that could impact the model’s diagnostic accuracy when applied to current imaging technologies. Secondly, this discrepancy may limit the model’s applicability across diverse clinical settings that utilize advanced mammographic techniques.

To address this limitation, we recommend further studies that validate the BCaXAI model using datasets derived from modern mammography technologies. Such validation would provide a more comprehensive assessment of the model’s robustness and its potential for integration into contemporary clinical workflows.

In conclusion, while our findings demonstrate the potential of the BCaXAI model for breast cancer diagnosis, the reliance on the CBIS-DDSM dataset—which reflects older film-based mammography technology—limits the generalizability of our results. Future research should validate the model using contemporary digital mammography datasets to enhance its applicability in current clinical settings.

## Figures and Tables

**Table 1:** The BCaXAI model metrics result in values.

Metric	Accuracy	Recall	F1 Score	Precision	AUROC Score
**Values**	0.9853	0.9853	0.9843	0.984	0.9933

**Table 2:** Performance comparison of the proposed BCaXAI model with ResNet50 and VGG16.

Metric	BCaXAI	ResNet50	VGG16
**Accuracy**	98.53%	95.20%	97.15%
**Recall**	98.53%	94.80%	96.82%
**Precision**	98.40%	95.50%	97.31%
**F1-Score**	98.43%	95.15%	97.06%
**AUROC**	99.33%	98.21%	98.75%

**Table 3:** Comparative Analysis of Recent Studies on DL Techniques for Breast Cancer (BCa) Classification.

Paper	Year	Methodology	Dataset	Challenges	Results
[19]	2024	-DL models: ResNet50, VGG16, AlexNet -Image classification: normal, benign, malignant BCa categories	DDSM	Need for refining models with larger datasets. Developing interpretable models for trust and insights.	ResNet50 accuracy of 96.23%. VGG16 and AlexNet also performed well with accuracies above 95%.
[20]	2024	Models: ResNet 50, ResNet 101, VGG16, and VGG19 for identifying breast cancer	Breast Histopathology Images	Breast cancer prognosis involves complex patterns that may be subtle and require sophisticated models to capture effectively.	ResNet50, ResNet101, VGG16, and VGG19 Accuracy (92.2%)
[21]	2023	ResNet and ResNetV2 architectures with transfer learning and fine-tuning Fine-tuning significantly boosts model performance	-Ultrasound breast imaging dataset -Fine-tuning and transfer learning datasets	-Challenges in image interpretation and classification in ultrasound-based breast imaging. -Need for improved diagnostic accuracy to reduce delayed diagnoses.	-ResNet50V2 model achieved 99.11% accuracy in breast image classification. -Fine-tuning significantly boosted model performance in BCa diagnosis.
[22]	2023	Automated detection of BC is first performed on pre-processed mammogram images	CBIS-DDSM	Accuracy needs to be improved. Need to be generalized on more datasets	Accuracy (89.2%), Recall (87.9%), F1-Score (0.895), Precision (91.3%)
[23]	2023	Lightweight attention ResNet50 module Dual-activated attention mechanism with fully connected layers	BreakHis dataset of BCa histopathological images Contains 7909 images with benign and malignant tumors	Data imbalance in medical image classification Interpretability challenges in DL BCa classification	Outperforms conventional models, visual transformers, and large models. Achieves accuracies of 98.5%, 98.7%, 97.9%, and 94.3%.
[24]	2022	-Stacked ensemble of ResNet models (ResNet50V2, ResNet101V2, ResNet152V2) -Comparative experiments with XGBoost classifier on individual models.	CBIS-DDSM INbreast	-Sequential integration of tumor detection, segmentation, and classification. -Achieving better performance than the latest DL methodologies.	-Pathology classification accuracy: 95.13%, 99.20%, 95.88% -BI-RADS category classification accuracy: 85.38%, 99%, 96.08%
[25]	2024	XAI, Privacy, federated learning, SHAP values computed locally	-Wisconsin Diagnostic Breast Cancer Dataset,	Addressing privacy concerns in AI models	97.59% accuracy, 98.393% F1 score in Wisconsin Diagnostic Breast Cancer Dataset
[26]	2023	Weakly supervised 3D DL BCa classification and lesion localization in MR images	Dataset: INbreast dataset Dataset: DDSM dataset	-Dependence on Medical Professionals -Addressing Incorrect Diagnoses	Radiologist correct performance: 85.7% accuracy, 98.5% sensitivity, 59.4% specificity Erratum corrected the layout of Table 4 in the paper.
[27]	2023	U-Net Architecture with Adam Optimizer for segmentation. Grad-CAM for visualization in convolutional neural networks decision-making process.	The data were collected and compiled by academics in Cairo University, Egypt	Lackluster interpretations and visualization methods in BCa research. Need for appropriate tools for diagnosis to avoid casualties.	Achieved accuracy, recall, precision, IoU, and F1-score of 99.37%, 86.20%, 81.30%, 84.60%, and 83.70%.
[28]	2023	A pipeline that incorporates data augmentation and image improvement. Using an effective U-Net-based segmentation technique, RoI is extracted from mammograms.	CBIS-DDSM	Accuracy needs to be improved. Need to be generalized on more datasets System Complexity	accuracy of 86.71% and a recall of 91.34
[29]	2023	Incorporates handcrafted features (local binary pattern (LBP) and histogram of orientated gradients (HOG)) in addition to features extracted from CNNs.	CBIS-DDSM	Designing effective handcrafted features requires domain expertise System Complexity	Accuracy (91.5%)
[30]	2023	Creates a new model called EACO-ResNet101 by fusing the EACO method with the ResNet101 structure.	MIAS and DDSM (CBIS-DDSM)	Implementing the integration of DL models with metaheuristic algorithms can be complex and requires expertise in both areas	Accuracy (98.63%), precision (98.71%)

**Table 4:** Comparison between the proposed BCaXAI model with state-of-the-art techniques and the CBIS-DDSM dataset.

Reference	Accuracy	Recall	F1 Score	Precision
Vinta, S. R. et al. [19]	96.23%	---	---	---
Das, H. S. et al. [22]	89.20%	87.90%	0.895	91.3%
Asma Baccouche, et al. [24]	90.02%	---	---	---
Bouzar-Benlabiod, L. et al. [28]	86.71%	91.34%	---	82.42%
Sajid, U. et al. [29]	91.50%	---	---	---
Thirumalaisamy, S., et al. [30]	98.63%	---	0.98	**98.71%**
**Proposed** **BCaXAI**	**98.53%**	**98.53%**	**0.9843**	98.40%

## Data Availability

https://www.kaggle.com/datasets/awsaf49/cbis-ddsm-breast-cancer-image-dataset (accessed on 15 May 2024).
